# Properties of Biocomposites Produced with Thermoplastic Starch and Digestate: Physicochemical and Mechanical Characteristics

**DOI:** 10.3390/ma14206092

**Published:** 2021-10-15

**Authors:** Adam Ekielski, Tomasz Żelaziński, Pawan Kumar Mishra, Jacek Skudlarski

**Affiliations:** 1 Department of Production Engineering, Institute of Mechanical Engineering, Warsaw University of Life Sciences (SGGW), Nowoursynowska 164, 02-787 Warsaw, Poland; adam_ekielski@sggw.edu.pl (A.E.); jacek_skudlarski@sggw.edu.pl (J.S.); 2 Faculty of Business and Economics, Mendel University in Brno, 61300 Brno, Czech Republic; xmishra@mendelu.cz

**Keywords:** biocomposites TPS, digestate, mechanical properties, thermal analysis, zero waste

## Abstract

This paper presents the results of a study on the influence of the addition of digestate (DG) sludge from an agricultural biogas plant on the mechanical properties of the coating obtained from thermoplastic starch (TPS). The dried, fragmented digestate, some of which had previously undergone ultrasound treatment, is used in the study. Biocomposites are produced by the pouring method using Teflon moulds as matrices. The physicomechanical study included the determination of the basic parameters of the materials obtained. Strength parameters, the contact angle, thermogravimetric properties (TGA), colour and colour difference and moisture absorption are determined. Photographs of the surface of the samples are taken with a scanning electron microscope (SEM) as well. It is found that the addition of the digestate has an advantageous effect on improving the physical and mechanical parameters. In general, samples with digestate also have a higher strength compared to the pure TPS material. The highest tensile strength and Young’s modulus are found in samples with the 14 wt% addition of ultrasound-treated digestate. On the basis of this study, it can be concluded that the addition of digestate is a promising approach for the production of TPS biocomposites with superior mechanical properties.

## 1. Introduction

Today’s efforts to improve the environment around us force the implementation of new eco-friendly materials that are substitutes for traditional plastic [1]. At the same time, the increasing ecological awareness demands that activities aimed at improving the environment are well perceived by society and are feasible [2]. Today’s trends and the increasing demand for biodegradable products mean that current biomaterials are no longer just simple utility products. More and more research centres are focusing on producing biomaterials for structural applications. Such materials can be used in many industries, e.g., automotive (upholstery and car cabin finishing elements), agricultural (agro-textiles, films, packaging, disposable tarpaulins and mats), construction and furniture (boards, panels and structural beams), and others [3,4]. As these trends are now being strongly promoted, this requires the implementation of technologies for manufacturing such products on a global scale.

According to Angellier-Coussy et al. [5] and Rodriguez et al. [6], the rational use of food processing by-products for the production of new biodegradable materials is reasonable. However, as stated by Lisowski et al. [7], the essence of producing an innovative material is the combination of an appropriate composition of the mixture and the selection of appropriate parameters of the technological process for the technological process of biocomposite production. This often poses a major problem, which is why the quality of many processed materials are often unsatisfactory. It is also one of many limitations that stand in the way of the intensive development of biodegradable materials [8].

It is, therefore, advantageous to search for and test new interesting raw materials that will significantly improve the quality of new biodegradable products. One of the problems of the modern industry is the elimination of waste generated during production processes [9]. The doctrine of “zero waste” is quite well known; however, the next step in the development of this view of sustainable economy will be the use of potential waste to produce products with the highest added value [10]. Among the many materials that meet the rules of biodegradability, many have significant potential for use is digestate (DG), resulting from anaerobic digestion in agricultural biogas plants. This raw material is mainly a residue of non-degraded lignocellulose, the so-called biomass recalcitrance, [11] with a significant mineral content. The high content of mineral substances such as P, N and K causes the digestate to be used as an organic fertilizer. In addition to mineral compounds, the digestate consists of significant amounts of lignin, hemicellulose and cellulose [12]. A previous study of the composition of the digestate indicate that, depending on the input feedstock composition used in the biogas plant and the course of the anaerobic digestion process, the DM dry matter of the digestate may contain 10–24% of cellulose, 6–18% of hemicellulose and 17–21% of lignin [13]. The substrate material decomposition takes place with the help of enzymes in the process of hydrolysis. 

Since one of the most difficult processes in a biogas plant is the hydrolysis of lignin and its protective function limiting the decomposition of polysaccharides contained in the plant material, the composition of the digestate depends on the ratio of lignin to cellulose in the raw material supplied to the process [14]. As a result of lignin hydrolysis, it is degraded and the polysaccharide compounds not protected by lignin are decomposed in the fermentation process into simple monosaccharide substances undergoing further degradation. Renouard et al. [15] claim that the pre-treatment of cellulose-rich plant fibre can be particularly useful in the production of biocomposites with improved strength. 

One way of treating plant fibres can include the method of sonification, as indicated by studies [16]. Sonification is used to partially disintegrate the material and, thus, increase its active surface area. The effect of the disintegration of the material is to wash out the soluble fractions and increase the hydrophobicity of the fibres [17]. 

The cellulose in the digestate that is not decomposed during the anaerobic digestion process is, therefore, partially modified feedstock cellulose. The remaining lignocellulosic compounds have a developed surface area and increased hydrophobic properties compared to the raw feedstock too. 

Therefore, it can be assumed that the processes occurring during anaerobic digestion in plant materials can constitute a good pre-treatment of lignocellulosic materials and can enhance the effects of ultrasonic waves, allowing the formation of stronger bonds between cellulose fibres and the matrix material of the composite. Studies on the use of digestate as the filler for polyester resin [18] allow obtaining adequate mechanical properties of the composite, thus, created, even at 70% of digestate in the polyester matrix. The composite created in this way, due to the use of polyester, is only a partially biodegradable material. Recently, a large part of studies has been focused on obtaining low-cost fully compostable materials. This group includes mainly natural polymers [6,19,20]. Among natural polymers, starch has been considered as one of the most promising raw materials for manufacturing biocomposites. Native starch has a crystalline structure which is not suitable for utilization in an intact form. To address this issue, starch gelatinization with glycerol is one of the common ways that yields elastic material, named thermoplastic starch (TPS). It interferes with the hydrogen bonds in the starch chain and protects it against retro-gradation; hence, making it flexible. A major drawback of TPS is its relatively low mechanical strength and hydrophilic properties. Numerous works are underway to reinforce TPS structures with fibrous substances [5,17]. A preliminary study of the TPS matrix with the addition of wood lignocellulose was carried out in our previous study, where an improvement in the physical and mechanical properties of the TPS reinforced with wood dust was indicated [20]. Nevertheless, the introduction of raw lignocellulosic materials (LC) in an untreated form decreases the moisture resistance of the, thus, obtained TPS–LC composite. The digestate processed by sonification, characterised by a relatively high lignin content, can be a good and cheap material for reinforcing composite structures (TPS–DG). In the literature, there are no reports on the influence of filling the starch matrix with digestate on its mechanical properties.

Therefore, the aim of this study is to examine the influence of selected fractions of digestate (DG) sludge from agricultural biogas plants on the physicochemical and mechanical parameters of thermoplastic starch materials.

## 2. Materials and Methods

### 2.1. Materials

The following components were used to produce thermoplastic biocomposite materials: potato starch (PPZ Trzemeszno, Trzemeszno, Poland), purified vegetable glycerine (ERPOL, Warsaw, Poland), dried digestate obtained from agricultural biogas plant, distilled water (Table 1).

#### 2.1.1. Study of Digestate

Pre-dewatered digestate from three biogas plants was used for the study. Each biogas plant operated using a different input material. For each type of digestate, the moisture content and the lignin and cellulose contents in dry matter were determined. The study was carried out according to the methodology described by [21]. The digestate selected for the research consisted of: dry matter 39%, cellulose 20.3% DM, Lignin 24.8% DM.

#### 2.1.2. Pre-Treatment of Digestate

Before the study, all the digestate received from the biogas plant A was dried to a moisture content of 6%. The dried digestate was fragmented in an MKM 6000 impact mill (manufacturer: BOSCH, Gerlingen, Germany) and then sieved using an LPzE-2e sieve classifier (manufacturer: MULTISERW-Morek, Brzeźnica, Poland) to obtain two uniform size fractions, i.e., 0 mm > 0.1 mm and 0.1 mm > 0.5 mm. Then, 100 g sample was weighed out from each fraction of the digestate for pre-treatment with ultrasonic waves. The sample was placed in a container filled with 1 dm^3^ of distilled water at the temperature of 25 °C. After mixing the suspension with a mechanical stirrer for 20 min, the sample of the digestate was subjected to ultrasound for 20 min using an Inter Sonic 37P generator equipped with ultrasonic head with a frequency of f = 40 kHz (manufacturer: Intersonic, Olsztyn, Poland). The ultrasonic intensity was 11.5 W·cm^−3^ and was determined based on the relation previously described [22]. The material treated with ultrasonic waves was drained and then dried at the temperature of 104 °C for 24 h. In order to wash out the soluble fractions from the digestate samples, the part of the samples not treated with ultrasound was also immersed in distilled water for 20 min, stirred and then dried under similar conditions as the samples treated with ultrasonic waves.

### 2.2. Biocomposite Production

**Figure 1:**
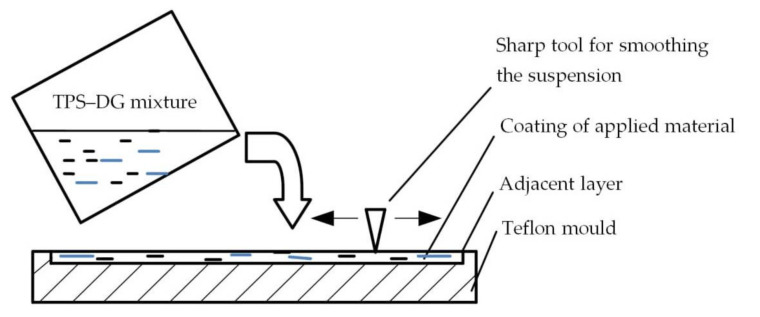
Example of TPS–DG film sample preparation in Teflon mould.

Table 1 shows the basic parameters of the mixtures used in the study. The preparation of the sample for the study consisted in mixing 50 g of potato starch, 25 g of vegetable glycerine and the addition of digestate of 7 wt% and 14 wt% in 500 mL of distilled water, respectively, which was the basic composition of the TPS biocomposite. The suspension prepared in this way was placed on the heater and stirred with a CAT 30 mechanical stirrer (manufacturer: CAT, Deerfield, IL, USA) at 300 rpm until the temperature of 85 °C was reached. After this time, the stirrer was turned off and the resulting suspension was poured onto Teflon moulds. The amount of the poured suspension was always constant and amounted to 250 g (±0.01 g). The thickness of the poured layer was determined using spacer plates (Figure 1) and was 1 mm. The top layer was levelled with a sharp tool until a surface roughness of Sr = +/−0.1 mm was achieved.

The prepared sample was then placed in a convection dryer and dried for 24 h at the temperature of 45 °C. The set drying temperature was set experimentally and caused the lowest risk of accidental cracking of the formed sheet of material. The inner edges of the mould were, additionally, composed of adhering material, which prevented the sample from curling during drying. The material (sheet of material) was gently removed from the mould and allowed to cool at room temperature. The thickness of the resulting sheet of material was 0.8 mm (±0.05 mm).

### 2.3. Mechanical Properties

The following parameters were tested: fracture strength, maximum elongation to fracture, Young’s modulus and puncture force. Paddles with a 6 mm × 80 mm diameter were prepared for breaking tests. Specimens of 100 mm × 40 mm were prepared for puncture force tests. An electronic calliper with an accuracy of 0.01 mm was used to measure the thickness of the sample at the site of analysis. The tests were performed according to the standards PN-EN ISO 527-1: 2020-01, PN-EN ISO 527-2: 2012 [23,24]. The AXIS 500 universal testing machine with an FA 200 N load cell with an accuracy of 0.01 N (manufacturer: AXIS, Gdansk, Poland) was used for the tests. The feed rate was 10 mm·min^−1^.The Young’s modulus (MPa) was calculated as the ratio of the stress difference *σ*_1_ and *σ*_2_, to the strain difference *ε*_2_ = 0.0025 and *ε*_1_ = 0.0005, *F*—force per specimen cross-section, *A*—initial cross-section of the matrix (mm). Formulas (1) and (2) used for calculations of Young’s modulus are presented below:

(1) $$\sigma =\frac{F}{A}$$

(2) $$E=\frac{\sigma_{2}-\sigma_{1}}{\varepsilon_{2}-\varepsilon_{1}}\;$$

where *σ*—the specified stress value, expressed in MPa; *F*—the corresponding force per section; *A*—the initial cross-section of the matrix, expressed in square millimetres.

### 2.4. Water Contact Angle

Wettability tests were performed using the “sitting drop” method described by Giri et al. [25]. The parameter analysed was the measurement of contact angle, which was calculated as the angle of inclination of the tangent to the projection of the droplet outline at the point of its contact with the tested surface. The measuring stand consisted of the following elements: digital camera A2500-14uc, 5 Mpix (16.2 Mpix matrix) with 18 mm–105 mm lens (manufacturer: Basler, Ahrensburg, Germany), adjustable measuring table, pipette for dispensing water drops of 15 µL. The photograph was taken max. 1 s after placing the drop on the material surface using the Pylon Viewer software (manufacturer: Basler, Ahrensburg, Germany). The contact angle was determined based on the technical analysis of the photographs using the Autodesk AutoCAD Mechanical v. 2019 software, product version: 23.0.46.0.

### 2.5. Colour

Colour tests were carried out by analysing photographs taken with an STX OPTA-TECH stereo microscope with 5 megapixel (Mpix) camera (manufacturer: OPTA-TECH, Warsaw, Poland). Before the tests, the camera was calibrated (white balance) with a Minolta plate no. 1863310. The photographed area was illuminated with an LED illuminator of light colour temperature of 7000 K (manufacturer: OPTA-TECH, Warsaw, Poland). For each test, samples were placed on the same white surface. The colour spaces L*, a*, b* were used for the colour measurements, where L*—brightness; a*—colour from green to magenta; b*—colour from blue to yellow. The CorelDRAW X7 Version 17.1.0.572 software (manufacturer: Corel Corporation, Ottawa, Canada) was used to read out the colour components (histogram function). Then, from the obtained results, the differences of colour were determined according to Equation (3):

(3) $$\Delta E={\left[{\left(\Delta L^{*}\right)}^{2}+{\left(\Delta a^{*}\right)}^{2}{\left(\Delta b^{*}\right)}^{2}\right]}^{1/2}$$

where ΔL*, Δa*, Δb* were the changes in colour values Δ*E* of samples with ultrasound-untreated digestate and ultrasound-treated digestate.

### 2.6. Scanning Electron Microscopy (SEM)

A HITACHI S-3400N Scanning Electron Microscope (Hitachi, Tokyo, Japan) was used to analyse the structure of the fabricated materials. Microscope settings: accelerating voltage 20 kV and in low vacuum 70 Pa. The surface of the fabricated samples was analysed.

### 2.7. Thermogravimetric Analysis

Thermogravimetric analysis (TGA) and differential thermal analysis (DTA) were performed on a Q50 TGA V20. 13., Build 39 (manufacturer: TA Instruments, New Castle, DE, USA). The following settings were used in the tests: heating rate 10 °C·min^−1^ from room temperature to the temperature of 700 °C, nitrogen flow rate (40 mL·min^−1^). A 50 mg sample was used for each measurement.

### 2.8. Differential Scanning Calorimetry (DSC)

The differential scanning calorimetry (DSC) was performed on a DSC 820 from Mettler Toledo (Schwerzenbach, Switzerland). The test consisted of placing a 4 mg sample in an aluminium crucible and analysing it at the temperature of −40–240 °C. The air flow rate was 60 mL·min^−1^, heating rate was 10 °C·min^−1^. The samples were analysed by one isothermal cycle and the data obtained were collected at the first heating cycle. DSC analyses were performed to compare the thermal properties of the three selected composites: pure TPS material, composite with digestate and composite with ultrasound-treated digestate.

### 2.9. Moisture Absorption

Before the tests, the material samples were cut into 1 cm x 8 cm pieces and dried in a convection dryer to a constant weight. Then, the samples were placed in a KBK-30 climate chamber (manufacturer: WAMED, Warsaw, Poland), relative humidity (RH) of 75% and at the temperature of 25 °C. During the test, the initial weight *W_o_* and the final weight *W_t_* were recorded for each measurement. The measurements were taken every one hour for 12 h. The percentage absorption was determined from Equation (4):

(4) $$Moisture\;absorption=\frac{W_{t}-W_{o}}{W_{o}}\times 100\%$$

### 2.10. Statistical Analysis

The results from the experimental tests (strength tests, colour change, contact angle) were processed using the Statistica 2013 software, version 13.3 (TIBCO Software Inc., Palo Alto, CA, USA). The mean values of the measured parameters were obtained from five samples (*n* = 5); then, the standard deviation (SD) was estimated. Normality of distribution was tested by the Shapiro–Wilk test. Significant differences between results were assumed to be significant at the confidence level of 95% (*p* < 0.05). These data were analysed using one-way analysis of variance (ANOVA) with Tukey’s post hoc test. Significant and non-significant differences were presented using lower and upper case letters placed next to the error bars. Different upper case letters, e.g., (C, B), indicate significant differences between the biocomposites (the same percentage of added digestate). Different lower case letters, e.g., (c, d), indicate significant differences between samples with different percentages of components. Lack of significance between homogeneous groups is indicated by, e.g., (b, b, or C, C), etc. For example (elongation stress) a statistically significant difference was found between DG7(05) and DG14(05), (a, b). Subsequently, a significant statistical difference was also found between the DG7(05) and DG7(05)U samples (D, C).

## 3. Results

### 3.1. Study of Digestate

The results of studies, presented in Table 2, indicated a significant influence of the type of substrate supplied to the biogas plant on the content of lignin and cellulose in the digestate. The lignin content in the digestate obtained was higher compared to those values reported in earlier studies [13]. This was probably due to the slightly different biogas production technology. The cellulose content was similar to the values reported in the cited publication [13]. Finally, the digestate with the highest fiber content was used for the study.

### 3.2. Mechanical Properties of TPS–DG

**Figure 2:**
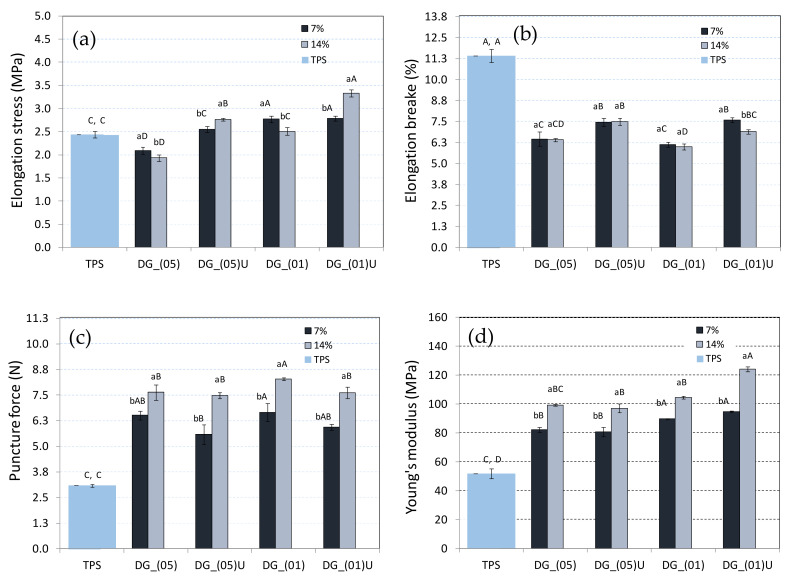
Strength tests of biocomposites: (**a**) elongation stress, (**b**) elongation break, (**c**) puncture force, (**d**) Young’s modulus (*p* < 0.05).

In general, all the strength analyses carried out indicated that the process parameters (proportion of digestate in the sample, type of fraction, or ultrasound treatment) could significantly affect the strength of TPS biocomposites. Large differences in results could also be observed when comparing the results to the parameters of TPS samples. Therefore, the digestate could be an interesting reinforcing addition for biocomposites based on thermoplastic starch. By analysing the results of the elongation stress tests of the samples (Figure 2a), it can be observed that the highest strength (3.3 MPa) was characteristic for the samples with 14 wt% of ultrasound-treated digestate. On the other hand, the samples with ultrasound-untreated digestate had the highest strength (2.7 MPa) at 7 wt% of digestate addition. This showed that the ultrasound treatment of the digestate has tangible benefits, such as improving the strength of the material while increasing the fraction of the digestate. This may also indicate a higher proportion of cellulosic components (released from the lignocellulosic material after ultrasound treatment) that better strengthen the TPS biocomposite. Cellulose is commonly added as a structural strengthening component of various materials [26]. Considering the above during sample preparation, the ultrasound-treated digestate interacted better with the suspension components, resulting in an increase in the strength of the TPS biocomposites. In this way, a higher elongation of the tensile materials (DG_(01)U and DG_(05)U) could also be explained, compared to the samples not treated with ultrasound (Figure 2b). An increased elasticity of cellulosic materials after ultrasound treatment was also indicated by Liu et al. [27]. It is worth noting, however, that the samples composed of pure TPS were characterized by a much higher elongation value (Figure 2b) compared to the samples created with the addition of digestate. However, this behaviour of the material was consistent with the results of other researchers’ studies [28].

Promising results were also obtained by testing the force needed to puncture the sample (Figure 2c). The tests showed that the strength of all materials increased with an increasing addition of the digestate. Compared to the strength of the TPS sample, the force needed to puncture the sample was up to 160% higher. This result was achieved with a 14 wt% addition of digestate DG14(01). In this case, the ultrasound treatment of the digestate had no significant effect on this strength parameter. It can also be observed in the graph that the 7 wt% addition of the digestate had an effect on increasing the force required to puncture the sample. It was also observed that the degree of fragmentation was significant only for sample DG14(01). Therefore, it can be concluded that, in the studied range of process parameters, each fragmented digestate improved the strength of TPS biocomposites. Comparing the obtained results to the parameters of similar products used commercially, it can be concluded that the results obtained were promising. For example, the strength of TPS films (puncture test), intended for padding strawberries, ranged from 8 to 26 N when punctured with a 5 mm mandrel [28]. In the case of our samples, the puncture test with a 2 mm mandrel was 8.2 MPa.

The studies of changes in the Young’s modulus (YM) values of the obtained composite material also confirmed the benefits of using the digestate as a reinforcing addition for thermoplastic starch biocomposites (Figure 2d). In particular, a large increase in all values was observed compared to the TPS sample (with a predominance for the samples with 14 wt% of the digestate). In this case, the highest YM values were achieved for the sample with 14 wt% of the digestate DG14(01)U. Additionally, comparing the obtained YM results to the studies of the above-mentioned films, the obtained results were satisfactory. According to studies [29], biodegradable TPS films can have a Young’s modulus of about 77–117 MPa. In the case of the analysed TPS samples, the Young’s modulus was in the range of 81–122 MPa.

### 3.3. Water Contact Angle

**Figure 3:**
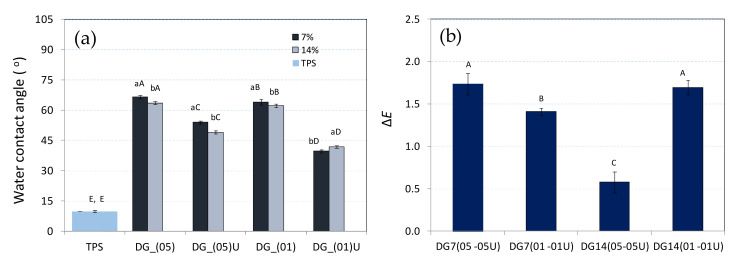
Contact angle charts and colour tests: (**a**) mean values of the changes in the contact angle. (**b**) Mean values of the difference in colour changes ΔE between the digestate and the digestate not treated with ultrasound.

By considering the water contact angle results (Figure 3a), it was found that all the values obtained were less than 90°. Therefore, it can be concluded that the obtained materials showed hydrophilic properties, as also indicated by Grylewicz et al. and Valencia et al. [30,31]. It was found that the highest contact angle (67°) was observed in the samples where the crude addition of the digestate was used. The observed contact angle for the sample composed of TPS was 10 degrees, adding digestate to the tested material caused a significant increase in the value of the contact angle (Figure 3a); as a consequence, the hydrophobicity of the material with digestate was increased compared to the reference material, which was pure TPS.

This increase could be related to the introduction of lignin, significant amounts of which can be found in the composition of the digestate [12,13]. It is related to the hydrophobic properties of lignin, its content in composite materials reduces the absorption properties of biocomposite materials, as indicated by the studies by Lisowski et al. [7]. It was also found that samples treated with ultrasound had a contact angle in the range of 40° to 52°. A decrease in this value indicated that ultrasound effectively degraded lignocellulose, which increased the access to hemicellulose. Hemicellulose is generally considered to be a hydrophilic material [32], which explains this course of the graph. It was also found that increasing the percentage of the digestate to 14 wt% usually caused a slight decrease in the contact angle value. These changes, however, were statistically insignificant in three cases. Comparing the obtained results to the studies of other authors, the most hydrophobic biodegradable material has a contact angle of 158° [33]. In contrast, the polylactic acid (PLA) commonly used for films has a contact angle of 75° [34].

### 3.4. Colour

**Figure 4:**
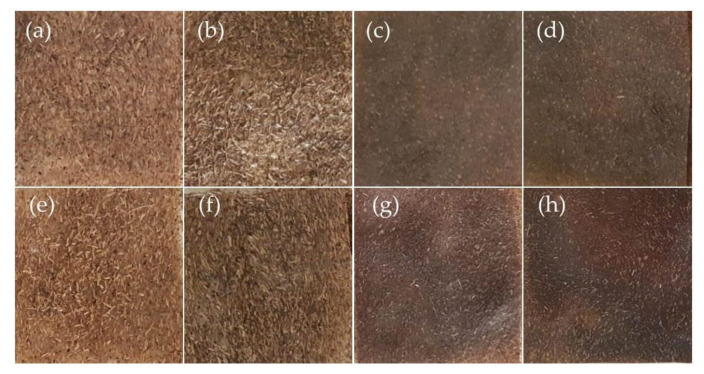
Images of materials obtained in the tests: (**a**) DG7(05); (**b**) DG14(05); (**c**) DG7(01); (**d**) DG14(01); (**e**) DG7(05); (**f**) DG14(05); (**g**) DG7(01); (**h**) DG14(01).

The analysis of the colour of biocomposites with the addition of the digestate showed that the samples were characterised by a dark colour with brown shades (Table 3). This colour was mainly due to the values of *a* and *b* parameters, which in the colour space L*, a*, b* range from −2.06 to 11.13. Generally, such a colour can also be present in the biocomposite, as indicated by Marcilhac et al. [35]. It was also found that the overall perception of colour can also be strongly influenced by the brightness of the product, which varied between 25.18 and 47.63 for samples with different proportions of digestate. For the TPS sample, the brightness was 76.17. By analysing the colour changes of the individual samples, it was found that the brightness of the materials decreased as the proportion of digestate in the sample increased. A similar trend was observed, also with increasing sample fragmentation. The darkest samples were, therefore, those from DG14(01) and DG14(01)U. At the same time, for these samples and all samples DG7(01,05)U, a decrease in brightness could be observed with the addition of the ultrasound-treated digestate. This may suggest a better dispersion between the particles of the digestate and TPS. Technologically, this may be important to obtain a uniformly coloured surface, especially using small additions of digestate to the TPS. The figure (Figure 3b) shows the overall colour differences between samples with and without ultrasound treatment of the digestate. It was found that the greatest differences were between samples DG7(05-05U) (Figure 3b). As the colour differences were mainly due to the fragmentation and incorporation of the particles into the TPS matrix, this indicated the high efficiency of the ultrasonic method. Similar results were also obtained for samples with 14 wt% digestate, DG14(01-01U). All colour changes can also be observed in the images of the obtained materials (Figure 4).

### 3.5. Scanning Electron Microscopy (SEM)

**Figure 5:**
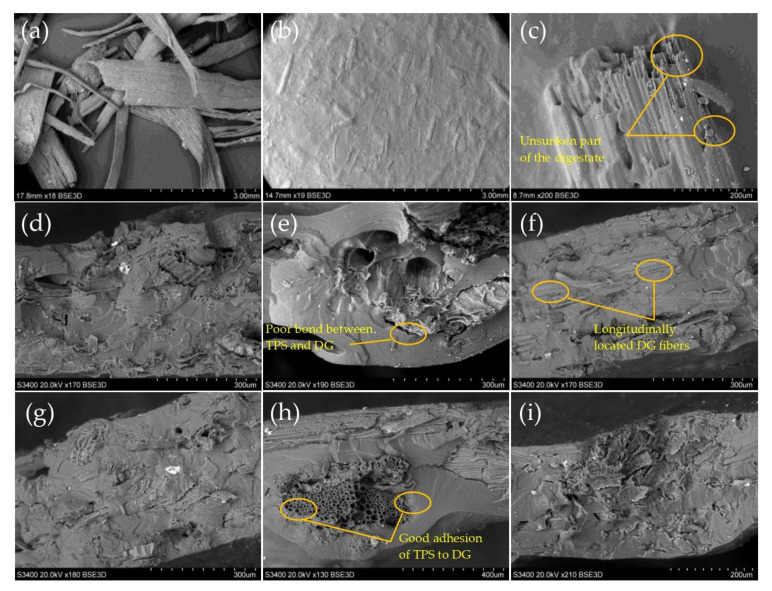
SEM microscopic images of digestate used in studies; (**a**) dried raw digestate; (**b**) exemplary digestate particle blended into TPS matrix; (**c**) surface view of composite obtained, 05 mm particles; (**d**) DG14(01); (**e**) DG14(05); (**f**) DG7(01); (**g**) DG14(01)U; (**h**) DG14(05)U; (**i**) DG7(01)U.

Figure 5a presents microscopic images of the dried raw digestate taken before undergoing the fragmentation process. A preliminary analysis showed that all particles had natural plant structures with visible fibrosis along the plants. The appearance of this structure indicated that there were still cellulose fibres inside them, which could be used to reinforce the TPS. Therefore, subjecting the digestate to the ultrasonic treatment and its further fragmentation would allow to obtain a material with a more developed surface, making it more useful for strengthening the structure of the biocomposite. In general, the ultrasound treatment of the digestate was aimed at disturbing the lignin and increasing the accessibility to the hydrophilic cellulose remaining in the digestate particles [36,37]. Such a treatment also facilitated the dispersion of particles during suspension preparation as indicated by the study of Osman et al. (2020) [38]. A surface analysis of the obtained samples showed that particles not treated with ultrasound were sometimes able to float to the surface of the material during drying (Figure 5b). Such a behaviour of the raw material could be a technological problem in the production of materials. The use of ultrasound allowed a good hydration of the raw material, which solved this problem. Figure 5c showed fine particles of the ultrasound-treated digestate, which were thoroughly blended into the TPS matrix Figure 5c. The positive feature of using ultrasound was, therefore, the improvement of the digestate properties, for a material with better physical properties (e.g., smooth surface). This problem was widely highlighted by researchers in various fields of science [39]. The floating of particles is often also associated with air storage inside the raw material particles. The elimination of this factor favourably influences not only the submersibility of the particles in suspension, but also the whole product. It is also an important factor from the ecological point of view [40].

Images of the breakthroughs of the samples were also taken (Figure 5d–i). On the basis of the performed analysis, it was found that DG particles (not treated with ultrasound) were less bound (mechanically) with TPS than particles treated with ultrasound. In the images, we can observe (Figure 5e) a small space indicating a poor adhesion of DG to TPS. There were no such areas in the image of the breakthrough of the DG14(01)U sample. In turn, in image (Figure 5f) DG7(01), longitudinally located DG fibres can be observed. Additionally, it may indicate poor sample binding at this point. There were no such areas in sample DG7(01)U. It can, therefore, be concluded that the ultrasound treatment had a positive effect on improving the structure of the produced biocomposites.

### 3.6. Thermogravimetric Analysis

**Figure 6:**
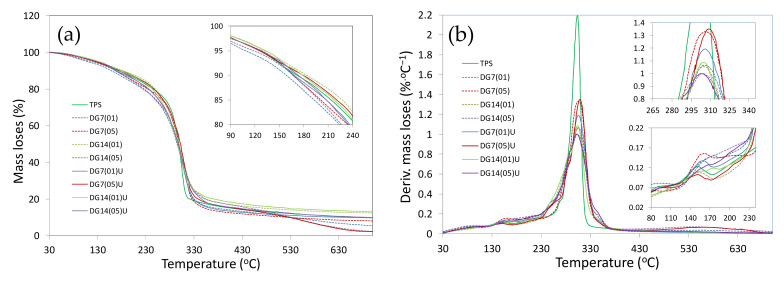
Thermogravimetric analysis of TPS biocomposites: (**a**) mass loses, (**b**) derivative mass change biocomposites TPS.

The results of the thermogravimetric analysis (TGA) and its derivative analysis (DTA) are presented in the graphs (Figure 6a,b). Then, in Table 3, the percentage mass losses determined for particular temperature ranges of the performed analysis were compared. Analysing the courses of graphs, it was observed that the mass loss of the pure thermoplastic material TPS was similar to the study [41] and proceeded in three stages. The first stage (30–170 °C) was mainly the evaporation of water and plasticizer. Thus, as indicated by [42], the onset of decomposition and weight loss resulted here mainly from the volatility of water and glycerol in the TPS. In the next stage (170–380 °C), at the temperature of 314 °C, a maximum degradation peak was observed, which was related to the decomposition of the starch chain [43]. The addition of the thermoplastic digestate to the material caused a significant reduction in the value of the maximum degradation peak. Five stages of degradation could also be observed on the thermograph runs. This was related to the introduction of the digestate, rich in lignocellulosic components, into the TPS. Thus, the weight loss at the temperature of 170–220 °C for samples with the addition of the digestate could be related to the onset of TPS starch transformations and to the softening of lignocellulosic components, mainly, cellulose as indicated by Kamdem et al. [44]. A further weight loss at the temperature of 220–260 °C could be related to the decomposition of hemicellulose. In this temperature range, in the DTA graph, a distinct shoulder was observed for all biocomposites from DG (Figure 6b). The next stage was the temperature range between 260 °C and 380 °C, where the changes on the thermographs were associated with cellulose degradation [45]. In this case, in the DTA graph for all biocomposites, the shoulder was visible at 350 °C. In this stage, the most resistant materials were samples with 14 wt% of DG14(05) and DG14(05)U digestate. This could be justified by the increased thermal resistance of lignin compared to the other lignocellulosic components [46]. This also explained the shift of the main shoulder on the DTA plot for TPS towards the temperature range of 310–330 °C. The last degradation stage (380–600 °C) was associated with the progressive depolymerisation of all components of the material samples. This stage was, mainly, the acetylated decomposition of high molecular weight components and the remaining transformations [47]. This explained the small peaks observed in the DTA graph in the temperature range of 450–600 °C. As mentioned in the literature review, digestate consists of residues of various plant particles. Hence, the differences. It was also observed that the highest degradation temperature of 131.8 °C, at which a 5% weight loss was observed, was found in samples DG14(01)U. In turn, in the sample marked as DG7(05)U, a weight loss of 50% was observed at the highest temperature of 301.9 °C.

### 3.7. Differential Scanning Calorimetry (DSC)

**Figure 7:**
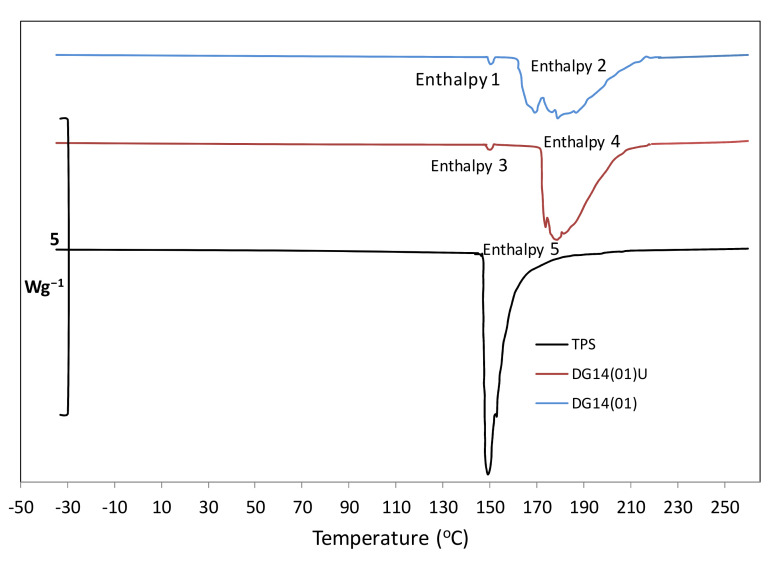
Plotted DSC thermographs of selected samples of TPS biocomposites.

Selected samples of biocomposites were analysed by the DSC method for their thermal response (Figure 7) and (Table 4). The aim of this test was to provide information on the interactions between the TPS starch components and the digestate and to determine the flow temperature of the material. It was found that the main endothermic phenomena in the analysed samples occurred in the temperature range of about 140–220 °C. It was observed that the pure TPS reached its endothermic maximum at a temperature of 139.53 °C. The tests were carried out on samples with the highest 14% addition of digestate DG14(01-01U) and compared to the TPS sample. The samples selected for testing had the best strength parameters (favourable for utility applications). The obtained results for the TPS were in agreement with the studies of other authors [48]. Two main endothermic areas can be observed in the endothermic plots of samples with the addition of digestate, presented in Figure 7. The first was the temperature range from 140 °C to 150 °C, which may have been due to the evaporation of water bound in the material. The second main area was related to the degradation of TPS starch and some of the components of the digestate. Since, in the composition of the digestate there were significant amounts of lignocellulosic components (lignin, cellulose and hemicellulose), the temperature range shifted towards higher temperatures (157.92–217.61 °C). The maximum endothermic peak was observed for samples with ultrasound-treated digestate (171.43 °C). Despite the addition of the digestate, which is rich in lignocellulosic components, at the set temperature of max. 260 °C, the exothermic peaks typical for these components were also not observed [49]. The composite samples containing the ultrasonically treated digestate were, therefore, more resistant to the influence of high temperature than the samples with the addition of crude digestate. Overall, this can be an advantageous feature of such materials as manufactured products decompose at higher temperatures. This may also be advantageous for the subsequent heat treatment and moulding, e.g., by extrusion or calendering.

### 3.8. Moisture Absorption

**Figure 8:**
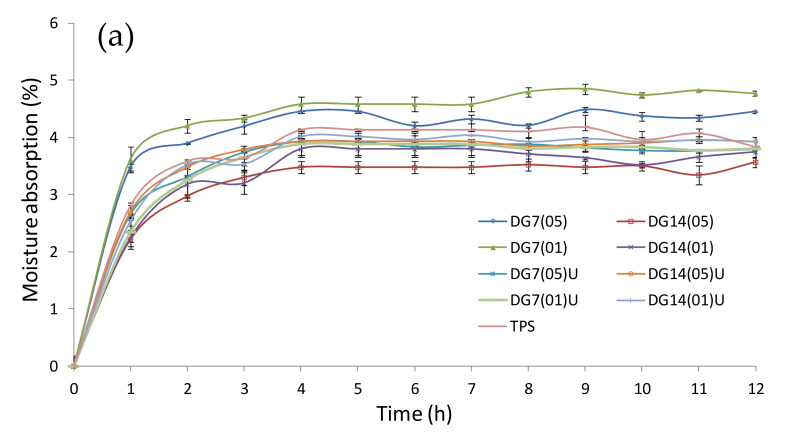

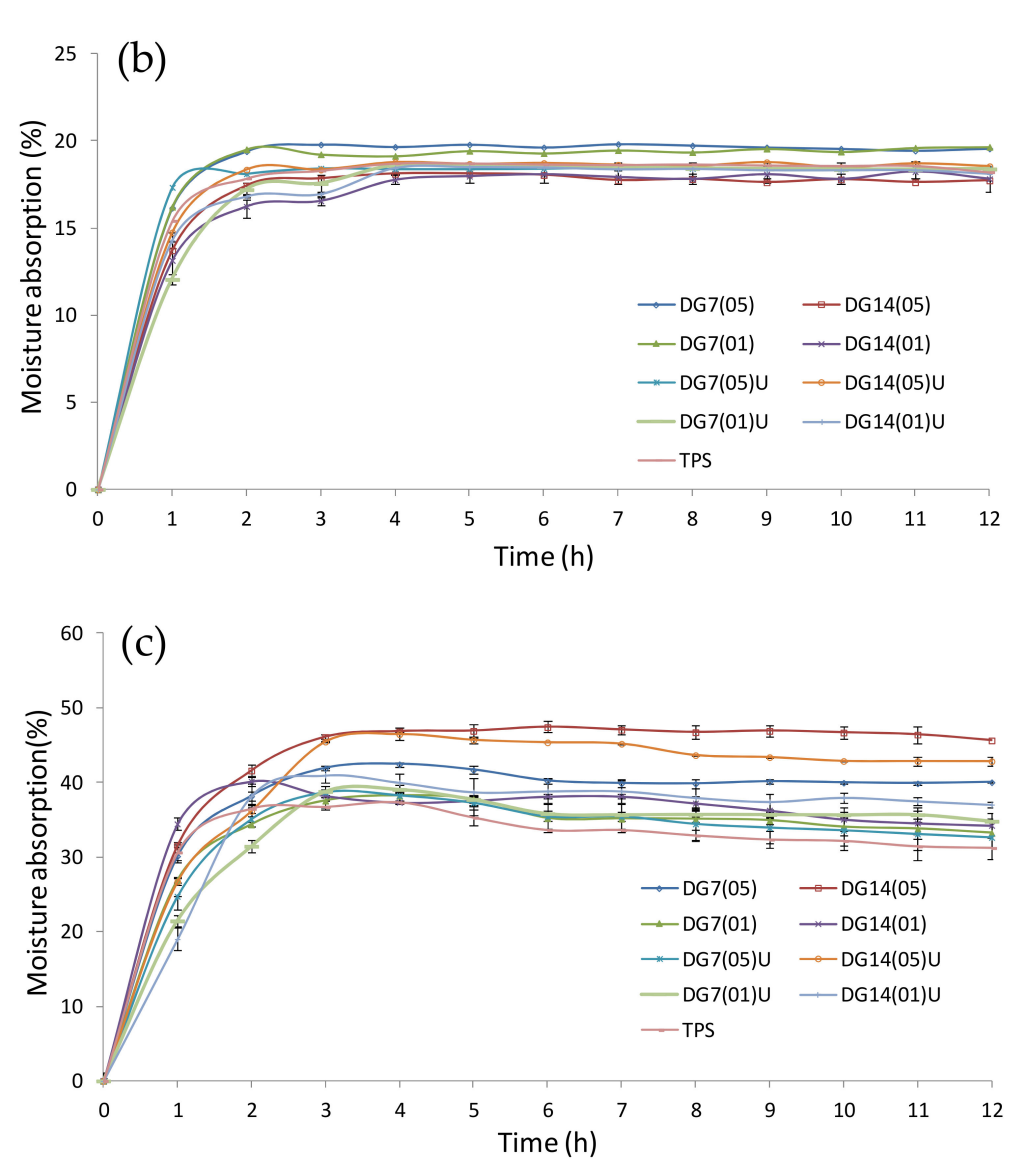
Course of changes in absorption of moisture from air by biocomposites. (**a**) Relative humidity, RH—50%; (**b**) relative humidity, RH—75%; (**c**) relative humidity, RH—90%.

An analysis of moisture absorption of the samples in the climatic chamber showed that the samples were sensitive to changes in air humidity. It was found that the samples were almost completely humidified already during the first three hours, which was indicated by significant changes in the moisture absorption of the obtained samples. The next 9 h of the sample stay usually caused only small percentage changes in moisture absorption. It was observed that during the first three hours of the sample stay in the atmosphere with a humidity of 50%, the moisture content could increase from 3% to 4.3% (Figure 8a). It was found that sample DG7(01) was the most sensitive to changes in humidity. In contrast, sample DG14(05) was the most resistant to moisture. The increase in the proportion of digestate in the sample and its degree of fragmentation had a significant effect on the moisture content of the sample. Such a trend of changes in the trend of the graphs could be observed for the other parameters of the climate chamber settings of 75% and 90% air humidity (Figure 8b,c). At air humidity of 75%, samples DG14(05) and DG14(01) were the most resistant to moisture. Samples DG14(05) and DG14(01) were moistened to the greatest extent. In this case, the obtained results oscillated in the range of 16–20% (moisture absorption), which was similar to the results of other authors [16]. It was interesting that the pure TPS was in the middle range of the obtained values. Then, at air humidity of 90%, a reversal of the trend for the samples to become moist was observed. In this case, samples DG7(01) were the most resistant materials. By analysing individual graphs, it can also be observed that samples in which the digestate was not treated with ultrasound better absorbed moisture from the air. This may be related to the heterogeneous dispersion of the suspension components, as also observed by Syafri et al. [16]. Another reason was the hydrophilic nature of the starch and lignocellulosic components [50]. In general, thermoplastic starch was characterised by high moisture absorption, as evidenced by our own studies and those of other researchers [51]. The addition of the digestate, which is rich in lignocellulosic components (especially lignin), may, therefore, increase the water absorption of such a material. However, it is interesting to note that the addition of 14 wt% of the digestate increased the moisture resistance of the material. In this case, the digestate probably acted as a natural barrier to moisture access to the pure TPS. From a technical point of view, this could be an advantageous feature of the materials obtained. Comparing the obtained results to other biodegradable materials, e.g., PLA had an absorbability of approx. 0.5% (air humidity 50%). To achieve similar results, the obtained materials should be further developed.

## 4. Conclusions

The studies showed that the addition of digestate (DG) to thermoplastic starch (TPS) made it possible to produce homogeneous biocomposites with a structure resembling a sheet or thick film. The studies presented that it was advantageous to use both a 7 wt% and 14 wt% digestate addition, which had an effect especially on improving the puncture force and Young’s modulus. The ultrasound-treated digestate made it possible to produce a biocomposite with the highest tensile strength (3.3 MPa) and the highest Young’s modulus (122 MPa) at the assumed process parameters. Regardless of the applied digestate, each addition increased the hydrophobicity of TPS biocomposites. The highest contact angle could be obtained by using ultrasound-treated unprocessed digestate as an addition. In order to obtain a better surface of the biocomposite (all particles embedded), it was more advantageous to use an ultrasound-treated digestate. It was also found that the ultrasound treatment of the digestate significantly affected the appearance of the materials obtained, as confirmed by delta E colour differences between the samples. The studies showed that materials with 14 wt% of digestate (0.1–0.5 mm fragmentation) were the most resistant to temperature changes. The obtained results confirmed the DSC analyses indicating a melt temperature shift towards higher temperatures. From the point of view of reprocessing the thermoplastic material, this was a favourable characteristic for such a material. The increase in the proportion of digestate in the sample and its degree of fragmentation had a strong influence on the moisture content of the TPS material. However, in order to limit this parameter, it was advantageous to use digestate with a fragmentation degree of 0.1–0.5 mm as an addition.

## Figures and Tables

**Table 1:** Parameters of the mixture for TPS–DG biocomposites.

Basic Components of TPS	Addition of Digestate DG to the Mixture (wt%)	Parameters of the Digestate (DG)		Acronym of the Obtained Film/Biocomposite TPS–DG
		Fraction (mm)	Ultrasonic Treatment	
Starch 50 g + glycerol 25 g	7	<0.1	Yes	DG_(01)U
	14	<0.1	Yes	DG_(01)U
	7	0.1–0.5	Yes	DG_(05)U
	14	0.1–0.5	Yes	DG_(05)U
	7	<0.1	no	DG_(01)
	14	<0.1	no	DG_(01)
	7	0.1–0.5	no	DG_(05)
	14	0.1–0.5	no	DG_(05)
	0	-	-	TPS

_—space for a percentage of digestate, e.g., DG7(01)U.

**Table 2:** Results of analyses of solid fraction of biogas digestate A, B, C. Basic component of material processed in biogas plant: plant A—corn silage; B, C—corn silage, cattle and pig manure, poultry manure. The content of components was determined for *p* <0.05.

Biogas Plant	Dry Matter (%)	Cellulose % DM	Lignin % DM
A *	39 ± 1.25	20.3 ± 0.58	24.8 ± 1.37
B	28 ± 2.63	17.2 ± 1.58	32.6 ± 2.98
C	29 ± 1.84	17.9 ± 2.66	30.2 ± 3.82

*—the digestate selected for the study.

**Table 3:** Average values of the L*, a*, b* colour components. Five replicate measurements (*p* < 0.05).

Sample	L*	a*	b*
DG7(01)	37.40 (±0.1)	3.52 (±0.03)	11.13 (±0.06)
DG7(01)U	36.26 (±0.53)	3.90 (±0.18)	10.41 (±0.44)
DG7(05)	46.09 (±0.18)	2.31 (±0.03)	10.50 (±1.11)
DG7(05)U	47.63 (±1.04)	2.54 (±0.21)	9.74 (±0.73)
DG14(01)	25.18 (±0.45)	1.11 (±0.13)	3.19 (±0.60)
DG14(01)U	24.47 (±0.75)	1.83 (±0.29)	4.55 (±0.31)
DG14(05)	30.36 (±0.87)	2.11 (±0.09)	6.96 (±0.30)
DG14(05)U	30.86 (±0.94)	2.14 (±0.30)	6.66 (±0.43)
TPS	76.17 (±0.70)	−2.06 (±0.13)	4.97 (±0.13)

**Table 4:** Analysis of DSC thermographs.

Measurement	DG14(01)		DG14(01)U		TPS
	Enthalpy 1	Enthalpy 2	Enthalpy 3	Enthalpy 4	Enthalpy 5
Onset (°C)	139.23	153.34	138.68	167.55	138.22
Peak Height (Wg^−1^)	0.15	1.01	0.15	1.54	3.75
Peak (°C)	140.2	170.92	139.65	171.43	139.53
Extrapol peak (°C)	140.31	157.92	139.73	171.08	138.76
Endset (°C)	141.97	217.61	141.84	196.85	150.52
Peak temperature range (°C)	1.39	36.06	1.59	22.51	7.80

## Data Availability

Not applicable.
